# Colorectal Cancer in Correlation With Clinicopathological Variables: The Effects of Hypoxia-Inducible Factor-1 Alfa or the InterLeukin-33 and Vascular Endothelial Growth Factor?

**DOI:** 10.7759/cureus.51658

**Published:** 2024-01-04

**Authors:** Hayat Ahmed, Mayada Ilias

**Affiliations:** 1 Department of Anatomy, Biology, and Histology, College of Medicine, University of Duhok, Duhok, IRQ; 2 Department of Pathology, College of Medicine, University of Duhok, Duhok, IRQ

**Keywords:** vegf, il-33, hif-1a, clinicopathological variables, colorectal cancer

## Abstract

Background

The hypoxia-inducible factor-1 alpha (HIF-1α) is believed to control angiogenesis and metabolism by upregulating hypoxia-induced genes, such as the interLeukin-33 (IL-33) gene and vascular endothelial growth factor (VEGF) gene. The study aimed to study the HIF-1α and its two hypoxia pathway genes; IL-33 and VEGF, together with the angiogenesis and correlate them with some prognostic clinicopathological features, separately and in combination to assess their dependency.

Methodology

This study included 87 colorectal cancer (CRC) cases, diagnosed between January 2019 and December 2022. Different prognostic clinicopathological features were examined and tissue microarray (TMA) slides were designed to carry out IHC for IL-33 and VEGF scoring in tumor cells, in addition to qualitative interpretation of VEGF expression in tumor vessels. Molecular analysis was performed for HIF-1α and all data were correlated to the clinicopathological features, separately and collectively, to assess the dependency of these factors.

Results

No statistical correlation could be seen among the IL-33, VEGF, and prognostic clinicopathological features. Whereas analysis of the HIF-1α alone showed significantly high mean expression in patients with distance metastasis and was increased with the increased involvement of the lymph nodes (LNs). However, when the HIF 1-α expression was correlated with the clinicopathological characteristics on the bases of VEGF and IL-33 expressions the significant association with metastasis disappeared in tumor cells and appeared only with the endothelium of the tumor angiogenesis. Moreover, the results conflicted with the LNs involvement.

Conclusions

These findings may suggest a role of HIF 1-α in the downstream regulation of biomarkers other than the VEGF and IL-33, which needs to uncover pathways and novel factors regulated by the HIF 1-α for the proinflammation and angiogenesis in malignancy.

## Introduction

The rapid proliferation of all cancer cells, including colorectal cancer (CRC), fosters oxygen deprivation, which stimulates a series of genes and sensitive factors that regulate the oxygen delivery pathways to adapt the hypoxia. One of the key players in this condition is the hypoxia-inducible factor (HIF) that changes the metabolism and promotes angiogenesis [1]. This factor consists of multiple oxygen-sensitive subunits, including HIF-1α, HIF-2α, and HIF-3α, in addition to β-subunit. Nowadays, accumulative evidence made it obvious that the maintenance and aggressiveness of any tumor are related to angiogenesis. In this concept, the HIF-1α is believed to control angiogenesis and metabolism by upregulating hypoxia-induced genes, such as the interLeukin-33 (IL-33) gene, vascular endothelial growth factor (VEGF) gene, and the glucose transporter (GLUT-1) gene [1-3]. The IL-33 belongs to the IL-1 family and is considered a proinflammatory cytokine [4]. While the VEGF marker is a pro-angiogenic factor that has been incriminated in the regulation of angiogenesis [5].

Contrary to the USA and some developed countries, in which the CRC decreases in mortality [6-9], in certain developing countries both the incidence and mortality of this disease are increasing, particularly in Iran, the nearest area to the Kurdistan Region /Iraq, which could be related to the Westernization of lifestyle [10]. However, the research in this field is still modest in these regions. In this study, molecular analysis was carried out for the HIF-1α in malignant cells within the tumor. The IL-33 and VEGF expressions were also estimated within the tumor cells and the expression of the later one was assessed within the vascularity of the tumor. We aimed to correlate all the results of the HIF-1α and its two-upregulating hypoxia-induced genes, to some prognostic clinicopathological features, separately and in combination to study the dependency of these variables to each other.

## Materials and methods

Patients and methods

This is a cross-sectional study, that included a total of 87 CRC cases, diagnosed between January 2019 and December 2022 at Vajeen Private Hospital and the Central Lab in Duhok Governorate, in Kurdistan Region, Iraq. Information and clinical data were taken from the pathological reports to access, in addition to the clinical information, the microscopical type of the operating surgeon, and the oncologist, to confirm certain clinicopathological variables like the staging. The paraffin-embedded blocks (PEB) were obtained from laboratory archives and new sections were made from the tumor area. The study included three parts: histopathology, immunohistochemistry (IHC), and molecular study.

Histological analysis

The histological reexamination was carried out by two pathologists separately, to confirm the findings in the pathology sheets and to assess special histopathological variables included in this research. The type grading and staging system were performed according to the recommendation of the American Joint Committee on Cancer of the CRC cases by Voge 2022 [11]. The inclusion and exclusion criteria for cases included in this study are shown in Table 1.

**Table 1:** The criteria for inclusion and exclusion of CRC cases in this study CRC: Colorectal cancer, PEB: Paraffin-embedded blocks, LNs: Lymph nodes

The inclusion criteria	The exclusion criteria
All the required clinicopathological data should be available in the pathology reports	Cases of CRC with missing information
Cases of CRC with biopsy as PEB	Small biopsies taken by colonoscopy
The biopsy should include the whole tumor and part of the colon	Biopsies taken without LNs or less than 12 LNs
Biopsies taken with at least 12 LNs	

IHC and tissue microarray (TMA) analysis

The slides were also examined to select the appropriate areas from the primary site, then the corresponding PEBs were marked for the desired areas and were used as donor PEBs to design TMA and build up TMA blocks (TMB) for IHC staining. The sections were mounted on ChemMate capilary Gap Plus slides (Grey Dako). Each TMB contains 12 samples (by obtaining three mm tissue) from each donor PEB, in addition to a positive and negative control samples in each run. The tissue was cored as cylinder tissue with a TMA instrument (Beecher Instruments, sun prairie, WI, USA) into a new TMB. About 4 μm thick sections were obtained from each TMB and mounted on a positive charge slide to establish microarray slides for IHC staining for the two markers: the IL-33 and VEGF. The Avidin Bioin Complex (ABC) detection system was used [12]. Automated IHC staining was used in Vajeen Private Laboratory, Duhok. Primary and secondary antibody kits for IL-33 and VEGF provided by the DAKO and SANTA CRUZ Company were used. Immune complexes were identified by using peroxidase reaction with DAB+ as chromogen (Envision+ detection system, K4006, Dako Corp, Carpinteria, CA).

For IL-33 expression, 10‑powered fields were chosen randomly, and about 100 cells in each field were counted. The score depended on both the percentage of immunoreactive cells and the staining intensity to obtain the overall score (OS) as follows: For percentage - 0-points 0% to 5%; 1-point 6% to 50%; 2-points >50%. For intensity - 1-point weak intensity; 2-point moderate intensity; 3-point strong intensity. Points from both intensity and percentage were added, and the OS was calculated then for statistical purposes the OS was modified as in Kamat 2009 into three groups: OS negative for 0 or 1, low expression for OS 2 or 3, and high expression for OS 4 or 5 [13]. The IHC stains of VEGF were evaluated according to the following semiquantitative grading score - negative, up to 10% IHC staining; low expression in > 10% to up to 50%; high expression in > 50%. Expression of VEGF in blood vessels was interpreted qualitatively (positive or negative). VEGF location of IHC staining was in membrane, cytoplasmic, and/or nuclei staining [14]. The results of the IHC staining were scored by two pathologists independently and blindly to the clinicopathological information of the patients.

Molecular analysis

For the molecular study, 79 samples with CRC tissue were used. The RNA was extracted from the tissues using the RNeasy mini kit (Qiagen, UK) and it was eluted in a volume of 20 μL of RNease free water as recommended by the manufacturer. The volume of RNA was obtained by the Nano-drop. The RNeasy PEB Kit Catalog number is 73504.

RNA was transferred to cDNA according to the manufacturer's protocol by using two-step RT-PCR PreMix and Power cDNA. All the reactions were set up on ice to minimize the risk of RNA degradation. The Glyceraldehyde 3-phosphate dehydrogenase (GAPDH) was used as reference genes “or as a Housekeeping gene (HKG).” Table 2 describes the primer properties that were used.

**Table 2:** Primers used in this study for the qPCR, for the target gene HIF-1α and Housekeeping gene; GAPDH. qPCR: Quantitative polymerase chain reaction, HIF-1α: hypoxia inducible factor -1 alpha, GAPDH: Glyceraldehyde 3-phosphate dehydrogenase, F: Forward. R: Reverse

Primer	Sequence (5ʹ –3ʹ)
HIF-1 α	(F): TATGAGCCAGAAGAACTTTTAGGC (R): CACCTCTTTTGGCAAGCATCCTG
GAPDH	(F): GTCTCCTCTGACTTCAACAGCG (R): ACCACCCTGTTGCTGTAGCCAA

Ethical approval

Ethical approval was obtained from the Ethics Committee of the Duhok Health Directorate as recommended by the Scientific Committee of the College of Medicine of Duhok University (Number 13072021-7-2, dated July 13, 2021), we also obtained informed consent from the respondents after explaining the objective of the study.

Statistical analysis

The data were analyzed using computerized data analysis. Cross tabulation test using the SPSS software (IBM Corp., Amonk, NY), with a P-value set at p < 0.001 indicates a significant difference. In the molecular data analysis, all the data were normalized to the housekeeping gene GAPDH. The Roto-gene software - Microsoft Excel sheet was used to indicate the Ct value. When the Ct has a negative value, the gene of interest is up-regulated because the change will be larger than 1, but when the Ct has a positive value, the gene is down-regulated, and the change is <1 according to Schmittgen and Livak [15]. The prevalence of HIF 1-α gene expressions in tumor cells of the CRC patients was examined in a Pearson chi-squared test, while their comparisons with different characteristics were examined in an independent t-test or ANOVA one-way. The pairwise comparisons were determined using a Tukey test. The significant level of difference was determined in a p < 0.05. The statistical calculations were performed using the JMP Pro 14.3.0 (https://www.jmp.com/en_us/home.html).

## Results

The study included 87 CRC patients; their ages ranged between 22 and 90 years. Their mean age was 57.85 ± 15.05. More than half of them were ≥ 60 years. Females represented > 56.3% of cases. The most common histological type was conventional adenocarcinoma, present in 87.36% of cases. Most of the patients (90.80%) presented for the first time at low grade, and in a relatively large percentage in T3 and T2 (55.17% and 26.44%, respectively). No lymph nodes (LNs) involvement was detected in 65.52% and 91.95% presented without secondaries at the first time of diagnosis, as seen in Table 3.

**Table 3:** General clinicopathological characteristics of CRC patients. CRC: Colorectal cancer, n: number, T: tumor size, N: nodes. M: metastasis

Characteristics (n=87)	Number	Percent
Age groups		
< 60	40	45.98
≥ 60	47	54.02
Gender		
Male	38	43.68
Female	49	56.32
Histological Type		
Conventional adenocarcinoma	76	87.36
Mucinous adenocarcinoma	9	10.34
Signet ring cell	2	2.30
Tumor Grade		
Low	79	90.80
High	8	9.20
T Category		
T 1	3	3.45
T2	23	26.44
T3	48	55.17
T4	13	14.94
N Category		
N0	57	65.52
N1	14	16.09
N2	16	18.39
Metastasis category		
M0	80	91.95
M1	7	8.05

**Figure 1:**
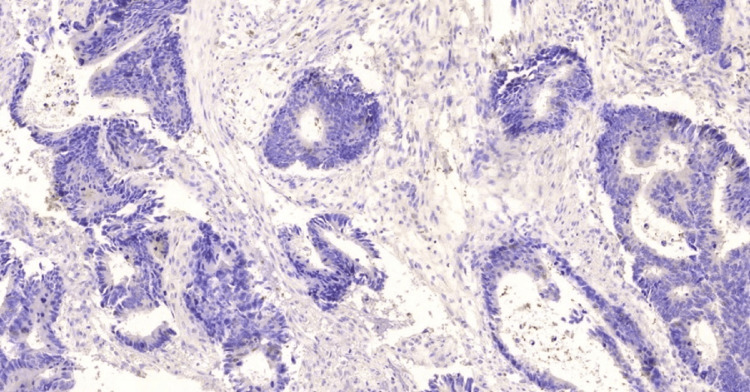
Negative expression IL-33 in conventional adenocarcinoma, 10x. IL-33: InterLeukin-33

**Figure 2:**
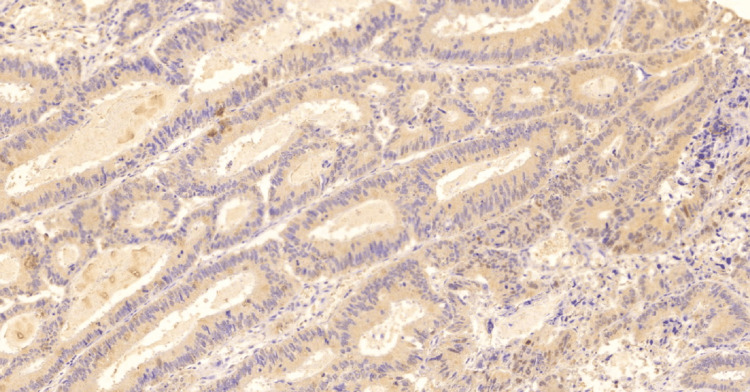
Low expression IL-33 in conventional adenocarcinoma, 10x. IL-33: InterLeukin-33

**Figure 3:**
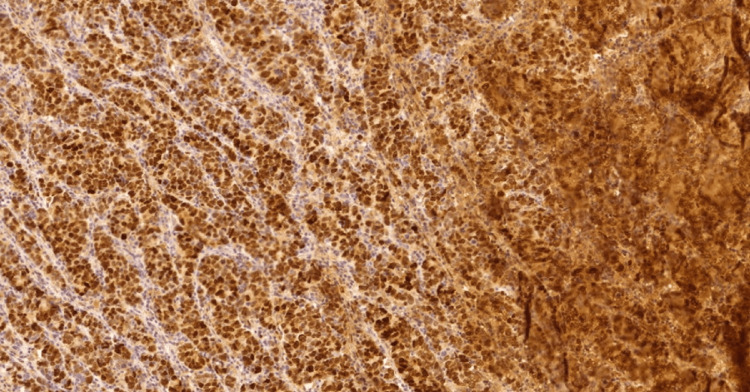
High expression IL-33 in conventional adenocarcinoma, 10x. IL-33: InterLeukin-33

**Figure 4:**
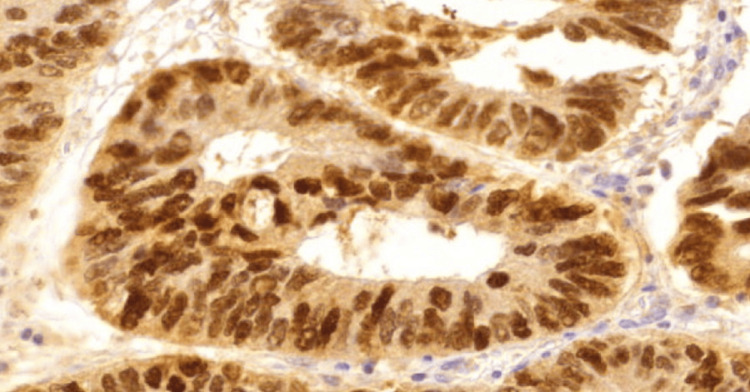
High expression IL-33 in conventional adenocarcinoma, 40x. IL-33: InterLeukin-33

The IL-33 expression in carcinoma cells was negative in 44 cases (50.57%) (Figure 1). Positive staining was detected in 43 cases (49.43); 25 cases of them had low expression (28.74) (Figure 2) and 18 cases had high expression (20.69) (Figures 3, 4). No statistically significant results could be seen between IL33 expression in carcinoma cells and any of the general clinicopathological characteristics (Table 4).

**Table 4:** Prevalence of IL-33 expression in tumor cells among CRC patients with general clinicopathological characteristics. *P-value <0.05 was considered significant IL-33: Interleukin-33, CRC: Colorectal cancer, n: number, T: tumor size, N: nodes. M: metastasis

Characteristics (n=87)	IL-33 expression in CRC n (%)			
	High expression 18 (20.69)	Low expression 25 (28.74)	Negative expression 44 (50.57)	P-value
Age				
<60	7 (17.50)	14 (35.00)	19 (47.50)	0.4692
≥60	11 (23.4)	11 (23.40)	25 (53.19)	
Gender				
Male	7 (18.42)	10 (26.32)	21 (55.26)	0.7414
Female	11 (22.45)	15 (30.61)	23 (46.94)	
Histological Type				
Conventional adenocarcinoma	14 (18.42)	23 (30.26)	39 (51.32)	0.6340
Mucinous adenocarcinoma	3 (33.33)	2 (22.22)	4 (44.44)	
Signet ring cell	1 (50.00)	0 (0.00)	1 (50.00))	
Tumor Grade				
Low	17 (21.52)	22 (27.85)	40 (50.63)	0.7704
High	1 (12.50)	3 (37.50)	4 (50.00)	
T Category				
T 1	1 (4.76)	8 (38.10)	12 (57.14)	0.1254
T2	11 (31.43)	10 (28.57)	14 (40.00)	
T3	5 (20.83)	7 (29.17)	12 (50.00)	
T4	1 (14.29)	0 (0.00)	6 (85.71)	
N Category				
N0	12 (21.05)	18 (31.58)	27 (47.37)	0.9286
N1	3 (21.43)	3 (21.43)	8 (57.14)	
N2	3 (18.75)	4 (25.00)	9 (56.25)	
Metastasis category				
M0	17 (21.25)	25 (31.25)	38 (47.50)	0.1226
M1	1 (14.29)	0 (0.00)	6 (85.71)	

From the total of 87 cases, 61 cases (70.11%) showed negative expression of VEGF, 20 cases (22.99%) had low expression, and only six cases (6.90%) had high VEGF expression in tumor cells. Again, no statistically significant results could be seen between VEGF expression in tumor cells and any of the general clinicopathological characteristics (Table 5).

**Table 5:** Prevalence of VEGF expression in tumor cells among CRC patients with general clinicopathological characteristics. *P-value <0.05 was considered significant VEGF: vascular endothelial growth factor, CRC: Colorectal cancer, n: number, T: tumor size, N: nodes. M: metastasis

Characteristics (n=87)	VEGF expression in tumor cells among CRC n (%)			
	High expression (n=6, 6.90%)	Low expression (n=20, 22.99%)	Negative expression (n=61, 70.11%))	P-value
Age				
<60	2 (5.00)	8 (20.00)	30 (75.00)	0.6294
≥60	4 (8.51)	12 (25.53)	31 (65.96)	
Gender				
Male	2 (5.26)	9 (23.68)	27 (71.05)	0.8678
Female	4 (8.16)	11 (22.45)	34 (69.39)	
Histological Type				
Conventional adenocarcinoma	5 (6.58)	81 (23.68)	53 (69.74)	0.7768
Mucinous adenocarcinoma	1 (11.11)	1 (11.11)	7 (77.78)	
Signet ring cell	0 (0.00)	1 (50.00)	1 (50.00)	
Tumor Grade				
Low	5 (6.33)	19 (24.05)	55 (69.62)	0.8421
High	1 (12.50)	1 (12.50)	6 (75.00)	
T Category				
T 1	1 (33.33)	1 (33.33)	1 (33.33)	0.2163
T2	0 (0.00)	4 (17.39)	19 (82.61)	
T3	5 (10.42)	12 (25.00)	31 (64.58)	
T4	0 (0.00)	3 (23.08)	10 (76.92)	
N Category				
N0	5 (8.77)	11 (19.30)	41 (71.93)	0.4970
N1	1 (7.14)	3 (21.43)	10 (71.43)	
N2	0 (0.00)	6 (37.50)	10 (62.50)	
Metastasis category				
M0	6 (7.50)	18 (22.50)	56 (70.00)	0.7298
M1	0 (0.00)	2 (28.57)	5 (71.43)	

The expression of VEGF in endothelial cells within the vascularity of the tumor was detected only in 39.08% of the cases. Although the negative expression was high in T2 and T4, the expression was equally distributed in T3, and the results were also statistically not significant for this variable as in other variables (Table 6).

**Table 6:** Prevalence of VEGF expression in endothelium among CRC patients with general clinicopathological characteristics. *P-value <0.05 was considered significant VEGF: vascular endothelial growth factor, CRC: Colorectal cancer, n: number, T: tumor size, N: nodes. M: metastasis

Characteristics (n=87)	VEGF expression in endothelium n (%)		
	Positive expression (n=34, 39.08%)	Negative expression (n=53, 60.92%)	P-value
Age			
<60	14 (35.00)	26 (65.00)	0.4718
≥60	20 (42.55)	27 (57.45)	
Gender			
Male	17 (44.74)	21 (55.26)	0.3410
Female	17 (34.69)	32 (65.31)	
Histological Type			
Conventional adenocarcinoma	30 (39.47)	46 (60.53)	0.8914
Mucinous adenocarcinoma	3 (33.33)	6 (66.67)	
Signet ring cell	1 (50.00)	1 (50.00)	
Tumor Grade			
Low	32 (40.51)	47 (59.49)	0.4420
High	2 (25.00)	6 (75.00)	
T Category			
T 1	2 (66.67)	1 (33.33)	0.0534
T2	5 (21.74)	18 (78.26)	
T3	24 (50.00)	24 (50.00)	
T4	3 (23.08)	10 (76.92)	
N Category			
N0	23 (40.35)	34 (59.65)	0.9408
N1	5 (35.71)	9 (64.29)	
N2	6 (37.50)	10 (62.50)	
Metastasis category			
M0	31 (38.75)	49 (61.25)	1.0000
M1	3 (42.86)	4 (57.14)	

The HIF 1-α mean expression in tumor cells was significantly high in patients ≥60 years (2.15) and in patients had distant metastasis (2.71). Although the mean expression was high in male (2.08) and in mucinous adenocarcinoma (2.62), these results were statistically insignificant. Similarly, the HIF 1-α expression was high in N1 (2.11) and higher in N2 (2.14) when compared with negative LNs involvement (1.70), these results were also statistically insignificant (Table 7).

**Table 7:** Comparisons of HIF 1-α expression in tumor among CRC patients with general clinicopathological characteristics. *P-value <0.05 was considered significant HIF-1α: hypoxia inducible factor-1 alpha, CRC: Colorectal cancer, n: number, SD, standard deviation, T: tumor size, N: nodes. M: metastasis

Characteristics (n=79)	HIF 1-α expression in tumor cells	
	Mean (SD)	P-value
Age groups		
< 60	1.59 (0.82)	0.0281*
≥ 60	2.15 (1.12)	
Gender		
Male	2.08 (1.17)	0.1935
Female	1.74 (0.87)	
Histological Type		
Conventional adenocarcinoma	1.85 (0.90)	0.2245
Mucinous adenocarcinoma	2.62 (2.27)	
Signet ring cell	1.81 (0.91)	
Tumor Grade		
Low	1.80 (0.92)	0.7384
High	1.92 (0.95)	
T Category		
T 1	2.16 (0.31)	0.1920
T2	2.25 (0.50)	
T3	1.67 (0.93)	
T4	1.93 (1.06)	
N Category		
N0	1.69 (0.95)	0.1690
N1	2.11 (0.35)	
N2	2.14 (0.86)	
Metastasis category		
M0	1.70 (0.89)	0.0053*
M1	2.71 (0.62)	

The correlation of HIF 1-α expression in tumor cells with the clinicopathological characteristics was carried out on the bases of IL-33 expression and the results are seen in Table 8. Although the HIF 1-α mean expression was significantly high in patients ≥60 years, no statistical significance could be seen between those with negative and positive IL-33 expression (wither the expression was high or low). Likewise, the HIF 1-α expression that have been significantly high in patients with distant metastasis showed no statistical differences among the subgroup of deferent IL-33 expression. However, patients with LNs involvement as N2 had significant high HIF 1-α expression only when the IL-33 expression was negative compared to low expression.

**Table 8:** Comparisons of HIF 1-α expression with the clinicopathological characteristics among sub-groups of CRC patients based on the IL-33 expression. Median (IQR): The values are in median (interquartile range) *P-value <0.05 was considered significant HIF-1α: hypoxia inducible factor-1 alpha, CRC: Colorectal cancer, IL-33: Interleukin-33, IQR: interquartile range, n: number, T: tumor size, N: nodes. M: metastasis

Characteristics (n=79)	HIF 1-α expression in Tumor			
	IL-33 expression in CRC Median (IQR)			
	High expression	Low expression	Negative expression	P-value
Age				
<60	1.164 (2)	2.088 (3.428)	1.993 (3.837)	0.3171
≥60	2.657 (2.669)	2.21 (2.453)	2.505 (1.646)	0.6679
Gender				
Male	2.094 (2.109)	2.059 (4.202)	2.359 (1.307)	0.4556
Female	1.795 (2.516)	2.187 (0.689)	2.21 (4.781)	0.4344
Tumor Grade				
Low	2.094 (2.367)	2.203 (3.308)	2.155 (1.583)	0.3326
High	1.164 (0)	1.033 (1.125)	3.035 (5.33)	0.1289
T Category				
T 1	0 (0)	1.892 (0)	2.296 (0.419)	0.2207
T2	0.179 (0)	2.195 (0.879)	2.636 (5.123)	0.2884
T3	1.945 (2.246)	2.117 (4.578)	1.99 (2.637)	0.5097
T4	2.657 (3.032)	1.863 (1.742)	2.555 (2.608)	0.9943
N Category				
N0	1.155 (2.827)	2.131 (2.415)	1.946 (1.203)	0.4681
N1	2.094 (2.07)	2.734 (3.532)	1.969 (4.974)	0.3305
N2	2.422 (1.493)	1.087 (0.871)	2.688 (1.054)	0.0273* (neg>low)
Metastasis category				
M0	1.795 (2.367)	1.976 (0.636)	1.899 (1.187)	0.9252
M1	2.657 (0)	0 (0)	2.887 (1.241)	0.6139

The correlation of HIF 1-α expression in tumor cells with the clinicopathological characteristics was also carried out on the bases of VEGF expression and there were no statistical differences with the age, sex, tumor grade and tumor size. Nevertheless, the HIF 1-α expression was significantly low when the N0 and N1 had negative VEGF expression. The results are seen in Table 9.

**Table 9:** Comparisons of HIF 1-α expression with the clinicopathological characteristics among sub-groups of CRC patients based on the VEGF expression. Median (IQR): The values are in median (interquartile range) *P-value <0.05 was considered significant HIF-1α: hypoxia inducible factor-1 alpha, CRC: Colorectal cancer, VEGF: vascular endothelial growth factor, IQR: interquartile range, n: number, T: tumor size, N: nodes. M: metastasis.

Characteristics (n=79)	HIF 1-α expression in Tumor			
	VEGF expression in CRC Median (IQR)			
	High expression	Low expression	Negative expression	P-value
Age				
<60	1.058 (1.085)	2.461 (3.592)	1.899 (1.42)	0.0934
≥60	1.479 (7.117)	1.99 (1.891)	2.657 (1.238)	0.3799
Gender				
Male	1.511 (1.989)	2.457 (2.793)	2.094 (1.491)	0.7228
Female	1.027 (6.891)	2.187 (1.884)	2.203 (2.422)	0.4721
Tumor Grade				
Low	0.516 (1.827)	2.426 (1.864)	2.179 (1.422)	0.0834
High	8.805 (0)	1.164 (0)	2.102 (1.721)	0.2826
T Category				
T 1	2.505 (0)	1.892 (0)	2.086 (0)	0.3679
T2	0 (0)	4.242 (9.009)	2.203 (1.26)	0.5307
T3	0.516 (4.976)	2.422 (1.892)	2.09 (3.476)	0.2902
T4	0 (0)	2.734 (1.86)	2.555 (1.823)	0.3036
N Category				
N0	0.516 (1.827)	2.21 (4.666)	1.946 (1.089)	0.0219* (neg
N1	8.805 (0)	2.492 (0.547)	1.852 (1.385)	0.0219* (neg
N2	0 (0)	1.797 (2.708)	2.555 (1.156)	0.8706
Metastasis category				
M0	1.058 (3.74)	2.04 (1.394)	1.923 (1.134)	0.5507
M1	0 (0)	2.523 (1.066)	2.719 (1.082)	0.6139

The correlation of HIF1-α expression in tumor cells with the clinicopathological characteristics was carried out on the bases of VEGF expression in endothelial cells in tumor vessels and the results are seen in Table 10. No statistical difference could be seen with the age, sex, tumor grade and tumor size, however, patients presented with LNs involvement in N1 had statistically higher HIF1-α expression (2.492) when the VEGF expression in endothelium was positive, but this result was not seen for N2 (1.797). Moreover, patients presented with metastasis (M1) had the highest HIF1-α expression (3.056) when the VEGF expression in endothelium was positive. This result was statistically high if compared with patients presented without metastasis or M0 (1.563) but not with those of M1 and negative VEGF expression in endothelium (2.657).

**Table 10:** Comparisons of HIF 1-α expression with the clinicopathological characteristics among CRC patients based on the VEGF expression in endothelium. Median (IQR): The values are in median (interquartile range) *P-value <0.05 was considered significant HIF-1α: hypoxia inducible factor-1 alpha, CRC: Colorectal cancer, VEGF: vascular endothelial growth factor, IQR: interquartile range, n: number, T: tumor size, N: nodes. M: metastasis.

Characteristics (n=79)	HIF 1-α expression in Tumor		
	VEGF Expression in Endothelium Median (IQR)		
	Positive expression	Negative expression	P-value
Age			
<60	2.086 (1.42)	2.457 (2.635)	0.8021
≥60	2.657 (1.261)	2.187 (2.019)	0.8289
Gender			
Male	1.973 (1.734)	2.492 (1.765)	0.1082
Female	2.453 (2.539)	1.679 (1.673)	0.0995
Tumor Grade			
Low	2.155 (1.18)	2.422 (1.383)	0.8705
High	2.102 (3.018)	2.391 (2.453)	0.7389
T Category			
T2	2.003 (3.626)	2.187 (0)	0.2443
T3	2.09 (2.841)	2.457 (2.141)	0.8233
T4	2.657 (0.966)	1.953 (1.562)	0.4132
N Category			
N0	1.968 (1.782)	1.899 (1.938)	0.8994
N1	1.852 (1.672)	2.492 (3.394)	0.0228*
N2	2.453 (1.246)	1.797 (2.038)	0.9529
Metastasis category			
M0	2.086 (1.312)	1.563 (1.332)	0.3592
M1	2.657 (1.204)	3.056 (0.898)	0.1840

## Discussion

Accumulative evidence points to a close relation between tumor hypoxia signaling and the angiogenic process depending on HIF-1α transcription of ILs, VEGF, and other factors. Since antiangiogenic therapy response and resistance depend on the understanding of this complex interaction [16,17], more weight should be lent to investigate the dependency of each factor and to clarify their prognostic values. As CRC had been considered the third cancer-related death in both sexes [7], this cancer had been chosen in the current study to investigate this complex relationship and its effects on the prognostic clinicopathological variables. Unlike the stable DNA, handling the unstable RNA to assess the HIF 1-α required tissue stored in PEB for not more than four years, which limited the number of cases.

Around the world, CRC varies in its incidence, mortality, and prognostic variables. These variations seem to be constantly changing, for instance, the age of occurrence and mortality are decreasing in certain years and countries and increasing in others [6,7]. The mean age of CRC cases in this study was 57.85, more than half of them were ≥ 60 years. Unexpectedly, the females were diagnosed more than the males. Recently in the United States, the incidence patterns were similar by sex. However, the age declined from 2014 to 2018 by nearly 2% per year in people ≥ 50 years but increased by about 1.5% per year in people younger than 50 years. This increase was also seen in several high-income countries [7,18]. Although all the included cases had at least 12 excised LNs and all cases have been examined by two pathologists, only 34.48% of cases had LN involvement, additionally, 8.05% had metastasis at the time of diagnosis. In a large cohort study that included 56,747 CRC patients from the Surveillance, Epidemiology, and End Results (SEER) database, the median age was 66.0 (which was higher than that in this study). The females were affected more than the males (51.5%) whereas, 59.5% of the patients had LN involvement at the time of diagnosis [19].

In this study, the IL-33 expression, in carcinoma cells, was positive in 49.43% (20.69% had high expression and 28.74% had low expression) while the VEGF expression was positive in 29.89% (6.90% had high expression and 22.99% had low expression). In endothelial cells, the VEGF expression was positive in 39.08% of the cases. No statistically significant results could be seen between these expressions and any of the prognostic clinicopathological characteristics. In this concept, it was stated that the IL-33 mediates its functions via a heterodimeric receptor complex and a broad range of actions affecting VEGF, angiogenesis, and metastasis, in other words, they contribute to different pathways and interaction [20,21]. Studies carried out in the past decade concluded that IL-33 either promotes or inhibits CRC in different settings. Emerging studies of IL-33 in mice also demonstrated contradictory results of promotion or inhibition [22-24]. Furthermore, Cui et al. found the expression of IL-33 was higher in early colonic adenomas when compared to adenocarcinoma [25]. Others have found that decreased IL-33 expression is associated with advanced human CRC, and increased expression showed reduced vascular invasion and LN metastasis [23]. From this debate, one can propose that studying additional factors may dissolve the intermingling condition. Therefore, the HIF 1-α, which controls angiogenesis by upregulating IL-33 and VEGF, was investigated separately and in combination with others. The HIF 1-α mean expression was significantly high in patients ≥60 years and cases of metastasis. In addition, the mean expression was high in N1 and higher in N2 when compared with those of N0 cases. In agreement with this result, many authors concluded that HIF-1 significantly influences cancer progression, from cell proliferation to angiogenesis and metastasis. Therefore, several inhibitors of HIF-1 have been suggested as therapeutic agents for different cancers, though, none of them have been translated into clinical therapy successfully [1,26]. However, in order to investigate the conflicting results of IL-33, VEGF, and angiogenesis, in this study the correlation of HIF 1-α expression with the clinicopathological characteristics was carried out on the bases of IL-33 and VEGF expression in tumor cells as well as VEGF expression in the endothelium of tumor. The results found that the significantly high HIF 1-α expression that has been seen in M1 (when analyzed alone), disappeared when analyzed on the basis of the subgroups of IL-33 and VEGF expression. Nevertheless, the conflict results in LNs involvement (for HIF 1-α, IL-33, and VEGF) and needs further analysis of additional factors or pathways. Similarly, several studies pointed to the wide, complex role of HIF 1-α in cancer including various interactions with growth factors and growth factor receptors [27], several effects on glycolytic enzymes such as the lactate dehydrogenase, promoting the production of ATP to sustain cell proliferation [28], regulating the natural killer cell mediating the antitumor effects and preventing cytotoxic response on tumor cells [29] and the upregulation of autophagy and escaping apoptosis particularly in CRC [30]. Hence the HIF-1 may affect cancer cell survival in different direct and indirect ways.

On the contrary, the correlation of HIF1-α with the VEGF expression in endothelial cells of angiogenesis showed that patients presented with metastasis had the highest HIF1-α expression (3.056) when the VEGF in endothelium was positive. In support of this finding, Jing et al. concluded that hypoxia is an important hallmark of the cancer microenvironment and angiogenesis because tumors develop as the result of transient fluctuations in the blood flow [2]. Efforts must be intensified to implicate the HIF-1 inhibitors in specific cancer therapy. Furthermore, it has been found that in various types of cancers, the development of resistance to chemo and radiotherapy is associated with overexpression of the HIF-1α [3]. Although the total number of patients included in this study was considered satisfactory, few variables or categories were detected in small numbers, making the correlation of these particular variables difficult. Therefore, larger studies with a deeper understanding of HIF-1 regulation and path in multiple cancer progression would improve the treatment and outcome of these cancers.

Limitations

The relatively small number of certain variables or categories like the small number of patients presented at high grade and in T1 or with special histological type, like the signet ring type, made the correlation uncertain. Therefore, a larger sample of patients may improve the statistical correlation. In addition, handling the unstable RNA forced the authors to select the more recently diagnosed patients, which limited the number of cases.

## Conclusions

The analysis of HIF-1α alone showed a more significant correlation with certain prognostic features of CRC than when HIF-1α was analyzed on the basis of VEGF and IL-33 expressions in tumor cells. However, the HIF-1α correlated more on the basis of VEGF in the endothelium of the tumor angiogenesis. This may support the belief that the HIF-1α may control angiogenesis and other metabolism by upregulating genes other than the IL-33 and the VEGF, and it regulates different paths in more complex actions that need to test the dependency of each biomarker, since this may improve the HIF-1 inhibitors used in specific cancer therapy.

## References

[1] Bui BP, Nguyen PL, Lee K, Cho J (2022). Hypoxia-inducible factor- 1: a novel therapeutic target for the management of cancer, drug resistance, and cancer-related pain. Cancers (Basel).

[2] Jing X, Yang F, Shao C, Wei K, Xie M, Shen H, Shu Y (2019). Role of hypoxia in cancer therapy by regulating the tumor microenvironment. Mol Cancer.

[3] Fang C, Dai L, Wang C (2021). Secretogranin II impairs tumor growth and angiogenesis by promoting degradation of hypoxia-inducible factor-1α in colorectal cancer. Mol Oncol.

[4] Cui G, Yuan A, Pang Z, Zheng W, Li Z, Goll R (2018). Contribution of IL-33 to the pathogenesis of colorectal cancer. Front Oncol.

[5] Wang Q, Gao J, Di W, Wu X (2020). Anti-angiogenesis therapy overcomes the innate resistance to PD-1/PD-L1 blockade in VEGFA-overexpressed mouse tumor models. Cancer Immunol Immunother.

[6] Li N, Lu B, Luo C (2021). Incidence, mortality, survival, risk factor and screening of colorectal cancer: a comparison among China, Europe, and northern America. Cancer Lett.

[7] Siegel RL, Miller KD, Fuchs HE, Jemal A (2022). Cancer statistics. CA Cancer J Clin.

[8] Brinzan CS, Aschie M, Cozaru GC, Deacu M, Dumitru E, Burlacu I, Mitroi A (2022). KRAS, NRAS, BRAF, PIK3CA, and AKT1 signatures in colorectal cancer patients in south-eastern Romania. Medicine (Baltimore).

[9] Arnold M, Sierra MS, Laversanne M, Soerjomataram I, Jemal A, Bray F (2017). Global patterns and trends in colorectal cancer incidence and mortality. Gut.

[10] Dolatkhah R, Somi MH, Kermani IA, Ghojazadeh M, Jafarabadi MA, Farassati F, Dastgiri S (2015). Increased colorectal cancer incidence in Iran: a systematic review and meta-analysis. BMC Public Health.

[11] Vogel JD, Felder SI, Bhama AR (2022). The American Society of Colon and Rectal Surgeons clinical practice guidelines for the management of colon cancer. Dis Colon Rectum.

[12] Bratthauer GL (2010). The avidin-biotin complex (ABC) method and other avidin-biotin binding methods. Methods Mol Biol.

[13] Kamat AA, Coffey D, Merritt WM (2009). EphA2 overexpression is associated with lack of hormone receptor expression and poor outcome in endometrial cancer. Cancer.

[14] Loureiro LVM, Neder L, Callegaro-Filho D, de Oliveira Koch L, Stavale JN, Malheiros SMF (2020). The immunohistochemical landscape of the VEGF family and its receptors in glioblastomas. Surg Exp Pathol.

[15] Schmittgen TD, Livak KJ (2008). Analyzing real-time PCR data by the comparative C(T) method. Nat Protoc.

[16] El Alaoui-Lasmaili K, Faivre B (2018). Antiangiogenic therapy: markers of response, "normalization" and resistance. Crit Rev Oncol Hematol.

[17] Abou Khouzam R, Brodaczewska K, Filipiak A (2020). Tumor hypoxia regulates immune escape/invasion: influence on angiogenesis and potential impact of hypoxic biomarkers on cancer therapies. Front Immunol.

[18] Siegel RL, Miller KD, Jemal A (2019). Cancer statistics. CA Cancer J Clin.

[19] Pei JP, Zhang CD, Fan YC, Dai DQ (2018). Comparison of different lymph node staging systems in patients with resectable colorectal cancer. Front Oncol.

[20] Jou E, Rodriguez-Rodriguez N, McKenzie AN (2022). Emerging roles for IL-25 and IL-33 in colorectal cancer tumorigenesis. Front Immunol.

[21] Liu X, Hammel M, He Y (2013). Structural insights into the interaction of IL-33 with its receptors. Proc Natl Acad Sci U S A.

[22] Maywald RL, Doerner SK, Pastorelli L (2015). IL-33 activates tumor stroma to promote intestinal polyposis. Proc Natl Acad Sci.

[23] Mertz KD, Mager LF, Wasmer MH (2016). The IL-33/ST2 pathway contributes to intestinal tumorigenesis in humans and mice. Oncoimmunology.

[24] He Z, Chen L, Souto FO (2017). Epithelial-derived IL-33 promotes intestinal tumorigenesis in Apc (Min/+) mice. Sci Rep.

[25] Cui G, Qi H, Gundersen MD (2015). Dynamics of the IL-33/ST2 network in the progression of human colorectal adenoma to sporadic colorectal cancer. Cancer Immunol Immunother.

[26] Masoud GN, Li W (2015). HIF-1α pathway: role, regulation and intervention for cancer therapy. Acta Pharm Sin B.

[27] Zhang Z, Yao L, Yang J, Wang Z, Du G (2018). PI3K/Akt and HIF‑1 signaling pathway in hypoxia‑ischemia (Review). Mol Med Rep.

[28] Naik R, Ban HS, Jang K (2017). Methyl 3-(3-(4-(2, 4, 4-Trimethylpentan-2-yl) phenoxy)-propanamido) benzoate as a novel and dual malate dehydrogenase (MDH) 1/2 inhibitor targeting cancer metabolism. J Med Chem.

[29] Chouaib S, Noman MZ, Kosmatopoulos K, Curran MA (2017). Hypoxic stress: obstacles and opportunities for innovative immunotherapy of cancer. Oncogene.

[30] Qureshi-Baig K, Kuhn D, Viry E (2020). Hypoxia-induced autophagy drives colorectal cancer initiation and progression by activating the PRKC/PKC-EZR (ezrin) pathway. Autophagy.

